# Mortality Risk for Docetaxel-Treated, High-Grade Prostate Cancer With Low PSA Levels: A Meta-Analysis

**DOI:** 10.1001/jamanetworkopen.2023.40787

**Published:** 2023-11-01

**Authors:** Brandon A. Mahal, Lucia Kwak, Wanling Xie, James A. Eastham, Nicholas D. James, Howard M. Sandler, Felix Y. Feng, Meryem Brihoum, Karim Fizazi, Christopher Sweeney, Praful Ravi, Anthony V. D’Amico

**Affiliations:** 1 Department of Radiation Oncology, University of Miami, Miami, Florida; 2 Department of Data Science, Dana-Farber Cancer Institute, Boston, Massachusetts; 3 Department of Surgery, Memorial Sloan Kettering Cancer Center, New York, New York; 4 Institute of Cancer Research, London, United Kingdom; 5 Department of Oncology, Royal Marsden NHS (National Health Service) Foundation Trust, London, United Kingdom; 6 Department of Radiation Oncology, Cedars-Sinai Medical Center, Los Angeles, California; 7 Department of Radiation Oncology and Urology, University of California, San Francisco; 8 Unicancer, Urogenital Tumor Study Group (GETUG), Paris, France; 9 Institute Gustave Roussy, Department of Cancer Medicine, University of Paris-Saclay, Villejuif, France; 10 South Australian Immunogenomics Cancer Institute, University of Adelaide, Adelaide, Australia; 11 Department of Medicine, Brigham and Women’s Hospital and Dana-Farber Cancer Institute, Boston, Massachusetts; 12 Department of Radiation Oncology, Brigham and Women’s Hospital and Dana-Farber Cancer Institute, Boston, Massachusetts

## Abstract

**Importance:**

Patients with high-grade prostate cancer with low levels of prostate-specific antigen (PSA; <4 ng/mL) are at high risk of mortality, necessitating an improved treatment paradigm.

**Objective:**

To assess for these patients whether adding docetaxel to standard of care (SOC) treatment is associated with decreased prostate cancer–specific mortality (PCSM) and all-cause mortality (ACM).

**Data Sources:**

PubMed search from 2000 to 2022.

**Study Selection:**

Five prospective randomized clinical trials (RCTs) performed in the US, France, and the United Kingdom evaluating SOC treatment with radiotherapy and androgen deprivation therapy (ADT) or with radical prostatectomy vs SOC plus docetaxel.

**Data Extraction and Synthesis:**

Individual data were included from patients with nonmetastatic prostate cancer, a PSA level of less than 4 ng/mL, and a Gleason score of 8 to 10. Patients initiated treatment between February 21, 2006, and December 31, 2015 (median follow-up, 7.1 [IQR, 5.4-9.9] years). Data were analyzed on December 16, 2022.

**Main Outcomes and Measures:**

Hazard ratio (HR) of ACM and subdistribution HR (sHR) of PCSM adjusted for performance status (1 vs 0 or good health), Gleason score (9 or 10 vs 8), tumor category (T3-T4 vs T1-T2 or TX), and duration of ADT (2 years vs 4-6 months).

**Results:**

From a cohort of 2184 patients, 145 patients (6.6%) in 4 RCTs were eligible (median age, 63 [IQR, 46-67] years). Thirty-one patients died, and of these deaths, 22 were due to prostate cancer. Performance status was 0 for 139 patients (95.9%) and 1 for 6 patients (4.1%). A reduced but nonsignificant risk of ACM (HR, 0.51 [95% CI, 0.24-1.09]) and PCSM (sHR, 0.42 [95% CI, 0.17-1.02]) was associated with patients randomized to SOC plus docetaxel compared with SOC. The risk reduction in ACM (HR, 0.46 [95% CI, 0.21-1.02]) was more pronounced among patients with a performance status of 0 and was significant for PCSM (sHR, 0.30 [95% CI, 0.11-0.86]).

**Conclusions and Relevance:**

Adding docetaxel to SOC treatment for patients who are in otherwise good health with a PSA level of less than 4 ng/mL and a Gleason score of 8 to 10 was associated with a significant reduction in PCSM and therefore has the potential to improve prognosis.

## Key Points

**Question:**

Is the addition of docetaxel to standard of care (SOC) therapy with radiation and testosterone suppression or radical prostatectomy associated with decreased risk of prostate cancer–specific and all-cause deaths in patients with high-grade, nonmetastatic prostate cancer with low prostate-specific antigen levels?

**Findings:**

In this meta-analysis of 4 randomized clinical trials (145 participants with prostate cancer), adding docetaxel to SOC treatment in patients who were in otherwise good health was associated with a significant reduction in death due to prostate cancer.

**Meaning:**

The poor prognosis of patients with high-grade prostate cancer and low levels of prostate-specific antigen levels may be improved with the addition of docetaxel to SOC treatment.

## Teaser

This meta-analysis evaluates whether adding docetaxel to testosterone suppression plus curative local treatment was associated with decreased mortality among patients with high-grade, localized or locally advanced, nonmetastatic prostate cancer with low serum levels of prostate-specific antigen (PSA).

## Introduction

Patients with high-grade prostate cancer and low levels of prostate-specific antigen (PSA) have an unfavorable prognosis when compared with patients with high-grade prostate cancer with elevated PSA levels.^1,2,3,4,5,6^ The reason for this observation is supported by the genomic characterization of these high-grade prostate cancers with low levels of PSA (<4 ng/mL; to convert to μg/L, multiply by 1.0) revealing genomic signatures that are associated with both resistance to testosterone suppression and a higher propensity to harbor micrometastatic disease at presentation.^7^ Therefore, for these patients, an improved treatment paradigm is needed.

Docetaxel has been shown to prolong overall survival (OS) in men with castrate-resistant and castrate-sensitive metastatic prostate cancer.^8,9,10,11^ However, 7 prospective randomized clinical trials (RCTs)^12,13,14,15,16,17,18^ that examined the role of adding docetaxel to National Cancer Center Network–defined standard of care (SOC) treatments^19^ have been completed and reported. The totality of evidence for use of radiotherapy and androgen deprivation therapy (ADT) with testosterone suppression alone or radical prostatectomy (RP) in patients with high-risk localized or locally advanced nonmetastatic prostate cancer suggests that treatment intensification with docetaxel does not uniformly improve treatment outcomes in these disease states.

In 1 of the 7 RCTs,^18^ a hypothesis-generating postrandomization analysis of OS stratified by PSA level was performed and yielded a hazard ratio (HR) of 0.33 among men with high-grade prostate cancer with low PSA levels when docetaxel was added to testosterone suppression for 6 months and primary radiotherapy. Furthermore, in a prior study of men with intermediate or high-risk nonmetastatic prostate cancer,^20^ only men with no or minimal comorbidity appeared to benefit from the addition of ADT to radiotherapy with respect to OS; those with moderate to severe comorbidity did not. Therefore, using individual patient data from the available RCTs from the Intermediate Clinical Endpoints in Cancer of the Prostate (ICECaP*)* Consortium database, we evaluated whether adding docetaxel to testosterone suppression plus curative local treatment (radiotherapy or RP) can reduce prostate cancer–specific mortality (PCSM) and all-cause mortality (ACM) in patients with localized or locally advanced nonmetastatic prostate cancer who have both a PSA level of less than 4 ng/mL and a biopsy Gleason score of 8 to 10 and are in otherwise good health with performance status defined as 0.

## Methods

This meta-analysis was not registered but is within the ICECaP collaboration with an institutional review board–approved protocol and a charter to regulate all secondary analyses; the statistical analysis plan (outlined below) was prespecified and followed for the current study. We used the checklist specifications for reporting of meta-analyses following the Preferred Reporting Items for Systematic Reviews and Meta-Analyses (PRISMA) reporting guideline.

### Patient Population and Treatment

Five RCTs that included 2597 patients treated with testosterone suppression plus radiotherapy or RP (SOC treatment) with or without docetaxel in the ICECaP database^13,14,16,17,18^ were eligible for study inclusion; the data in these studies were updated as reflected in the most recent date of publication. Two RCTs^12,15^ could have been helpful to answer the study question, but their data were not available.

**Figure 1.**
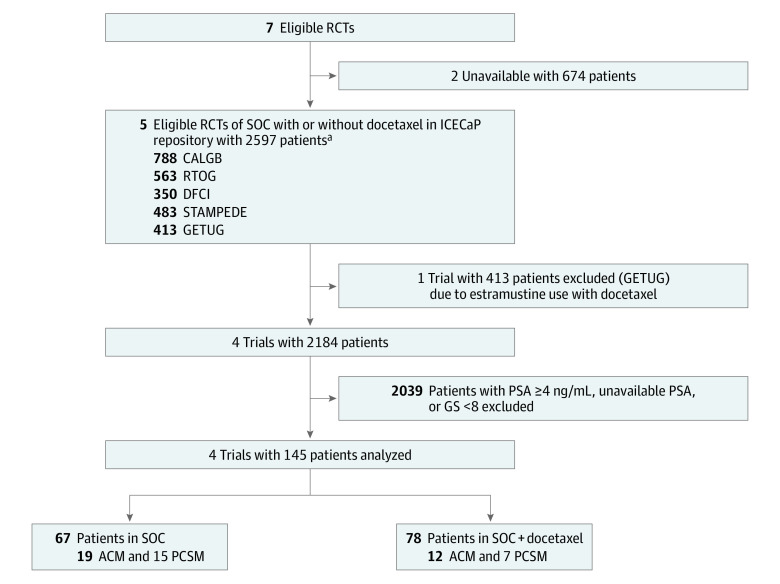
Study Flow Diagram The flow of identification, screening, and eligibility of the 145 patients included in the meta-analysis as well as the distribution of standard of care (SOC) or SOC plus docetaxel treatment and rates of all-cause mortality (ACM) and prostate cancer–specific mortality (PCSM) among the 145 study patients included in the meta-analysis. CALGB indicates Cancer and Leukemia Group B; DFCI, Dana-Farber Cancer Institute; GETUG, Urogenital Tumor Study Group; GS, Gleason score; ICECaP, Intermediate Clinical Endpoints in Cancer of the Prostate; PSA, prostate-specific antigen; RTOG, Radiation Therapy Oncology Group; and STAMPEDE, Systemic Therapy in Advancing or Metastatic Prostate Cancer: Evaluation of Drug Efficacy. ^a^We included 483 patients with M0 prostate cancer who were planned for or received radiotherapy in the STAMPEDE trial.

Treatment consisted of either RP alone or radiotherapy and ADT for 6 months to 2 years in the SOC group. For patients treated with radiotherapy, 3-dimensional conformal radiotherapy was primarily used, and inclusion of the pelvic lymph nodes in the radiation volume was generally performed for patients with Gleason scores of 8 to 10. In the docetaxel group of the RCT,^17^ where RP was the SOC treatment, patients also received 18 to 24 weeks of ADT. Patients with localized or locally advanced nonmetastatic prostate cancer who had a serum PSA level of less than 4 ng/mL and a biopsy Gleason score of 8 to 10 were further identified, totaling 150. The 5 eligible patients of the 413 enrolled in the Urogenital Tumor Study Group (GETUG)–12 RCT^16^ were excluded due to the use of estramustine with docetaxel, given that estramustine is no longer used in the treatment of prostate cancer after being shown to significantly increase the risk of thromboembolic events^21^ and therefore increase ACM. This led to a final study cohort of 145 patients as shown in Figure 1.

### Clinical Outcome Measures

All-cause mortality and PCSM were coprimary end points. All-cause mortality was measured from the date of randomization to death due to any cause, censored at the date of last follow-up in surviving patients. Prostate cancer–specific mortality was defined analogously to ACM, but death not due to prostate cancer was considered a competing risk. Cause of death was based on trial-defined events.

### Statistical Analysis

Data were analyzed on December 16, 2022. The distributions of the patient, cancer, and clinical factors including self-reported race as per institutional guidelines were enumerated according to randomized treatment group (SOC with or without docetaxel). For the purpose of illustration, the unadjusted distributions of ACM and PCSM were calculated by randomized treatment group using the 1 − Kaplan-Meier method^22^ and cumulative incidence function accounting for competing risks,^23^ respectively. These distributions were compared across randomized treatment groups using a log-rank^24^ *P* value for the end point of ACM and Gray test^23^ *P* value for the end point of PCSM.

To evaluate the treatment effect on the coprimary end points, an HR (with its 95% CI) was estimated for ACM using multivariable Cox proportional hazards regression,^25^ and a subdistribution HR (sHR) (with its 95% CI) was estimated for PCSM using multivariable Fine and Gray^26^ competing risk regression, with adjustment for performance status (1 vs 0 [reference]), biopsy Gleason score (9 or 10 vs 8), clinical T category (T3-T4 vs T1-T2 or TX), and duration of ADT (2 years [long-term] vs 4 to 6 months [short-term]). Duration of ADT was added as a covariate to the model given that long-term ADT has been shown to be superior to short-term ADT in the management of high-grade prostate cancer with respect to the end point of OS.^27^ Performance status of 0 (or otherwise good health) was defined as Eastern Cooperative Oncology Group, Zubrod, or World Health Organization performance status scores of 0. Performance status data were not available for patients enrolled in CALGB (Cancer and Leukemia Group B) 90203,^17^ and they were assigned a performance status of 0, given that they needed to be considered medically able to undergo an RP. A subgroup analysis was performed in the 139 patients with performance status of 0 to investigate whether the treatment effect of adding docetaxel to SOC treatment on the risk of PCSM and ACM was consistent with the results in the overall cohort. Given that only 6 patients had a performance status of 1, an interaction test between performance status and the docetaxel treatment effect was not possible.

A test for heterogeneity was not performed given that 1 clinical trial^14^ contributed only 9 patients to the study cohort. However, the study cohort was homogenous, with all patients having a PSA of less than 4 ng/mL and biopsy Gleason score of 8 to 10, and the randomized treatment groups were similar, consisting of SOC plus docetaxel vs SOC. These factors combined should minimize heterogeneity of the treatment effect on the end points of ACM and PCSM. All statistical tests were 2-sided, and the Bonferroni method^28^ was used for multiplicity adjustment of the 2 comparisons performed (ie, the entire study cohort and patients with performance status of 0) when calculating the unadjusted estimates of PCSM and ACM such that *P* < .025 (.05/2) was considered statistically significant. Median follow-up and associated IQR were calculated using the reverse Kaplan-Meier method.^29^ We used Stata/SE, version 16.1 (StataCorp LLC) for all statistical analyses.

## Results

### Patient Population, Treatment, PCSM and ACM Event Rates

As shown in Figure 1, of the 2184 men enrolled in the 4 eligible RCTs, 145 (6.6%; median age, 63 [IQR, 46-67] years) met the selection criteria of PSA of less than 4 ng/mL, biopsy Gleason score of 8 to 10, and no estramustine use and initiated treatment between February 21, 2006, and December 31, 2015. A total of 5 patients (3.4%) were Asian, 6 (4.1%) were Black, 118 (81.4%) were White, 2 (1.4%) were of other race or ethnicity (including American Indian or Alaska Native and Hispanic or Latino), and 14 (9.7%) were of unknown race or ethnicity. A total of 67 patients were in the SOC group and 78 in the SOC plus docetaxel group; 139 (95.9%) had performance status of 0 and 6 (4.1%) had performance status of 1; 94 (64.8%) underwent RP and 38 (26.2%) had 2 years of ADT. The patient, cancer, and clinical factors were similar between the 2 randomized treatment groups as shown in Table 1.

**Table 1.** Distribution of the Patient, Cancer, and Clinical Factors Stratified by Treatment Group for the 145 Patients in the Study Cohort

Clinical characteristic	Treatment group^a^	
	SOC (n = 67)	SOC plus docetaxel (n = 78)
Race and ethnicity		
Asian	0	5 (6.4)
Black	0	6 (7.7)
White	57 (85.1)	61 (78.2)
Other^b^	0	2 (2.6)
Unknown	10 (14.9)	4 (5.1)
Year of randomization		
2006-2011	44 (65.7)	49 (62.8)
2012-2015	23 (34.3)	29 (37.2)
Age at randomization, median (IQR), y	65 (61-69)	62 (58-67)
ECOG performance status		
0	63 (94.0)	76 (97.4)
1	4 (6.0)	2 (2.6)
Biopsy Gleason score		
8	27 (40.3)	29 (37.2)
9	37 (55.2)	45 (57.7)
10	3 (4.5)	4 (5.1)
PSA level at randomization, median (IQR), ng/mL	2.6 (1.5-3.2)	2.5 (1.3-3.4)
Clinical tumor category		
T1-TX	13 (19.4)	16 (20.5)
T2	29 (43.3)	41 (52.6)
T3-T4	25 (37.3)	21 (26.9)
Primary local therapy		
External beam radiotherapy	25 (37.3)	26 (33.3)
Radical prostatectomy	42 (62.7)	52 (66.7)
Duration of ADT		
Short-term (≤6 mo)	49 (73.1)	58 (74.4)
Long-term (2-3 y)	18 (26.9)	20 (25.6)
Follow-up, median (IQR), y	7.3 (5.5-9.9)	6.6 (5.3-9.7)

Abbreviations: ECOG, Eastern Cooperative Oncology Group; PSA, prostate-specific antigen.

SI conversion factor: To convert PSA to μg/L, multiply by 1.0.

^a^ Unless otherwise indicated, data are expressed as No. (%) of patients.

^b^ Includes American Indian or Alaska Native and Hispanic or Latino.

During a median follow-up of 7.1 (IQR, 5.4-9.9) years (6.4 [IQR, 4.7-8.2] years for RP RCT; 10.1 [IQR, 8.6-11.5] years for radiotherapy plus ADT RCTs) among the 145 patients in the study cohort, 31 (21.4%; 19 in the SOC group and 12 in the SOC plus docetaxel group) died; of these deaths, 22 (71.0%; 15 in the SOC group and 7 in the SOC plus docetaxel group) were due to prostate cancer. Among the 139 patients with performance status of 0, 27 died (19.4%; 17 in the SOC group and 10 in the SOC plus docetaxel group); of these deaths, 19 (70.4%; 14 in the SOC group and 5 in the SOC plus docetaxel group) were due to prostate cancer.

### Risk of ACM and PCSM

After adjustment for covariates, a nonsignificant reduced risk of ACM (HR, 0.51 [95% CI, 0.24-1.09]) and PCSM (sHR, 0.42 [95% CI, 0.17-1.02]) was associated with patients randomized to the SOC plus docetaxel group compared with the SOC group (Table 2). The risk reduction in ACM (HR, 0.46 [95% CI, 0.21-1.02]) was more pronounced among men with performance status of 0 and significant for PCSM (sHR, 0.30 [95% CI, 0.11-0.86]) as shown in Table 3.

**Table 2.** Covariate-Adjusted HRs for Mortality in 145 Study Patients With Performance Status of 0 or 1

Covariate	End point	
	ACM, HR (95% CI)^a^	PCSM, sHR (95% CI)^b^
With vs without docetaxel	0.51 (0.24-1.09)	0.42 (0.17-1.02)
Performance status 1 vs 0	5.24 (1.48-18.58)	7.20 (1.27-40.86)
Clinical T category T3-T4 vs T1-T2 or TX	1.64 (0.78-3.48)	2.32 (1.04-5.22)
Gleason score 9-10 vs 8	2.20 (0.90-5.34)	3.14 (0.78-12.66)
ADT duration long-term vs short-term	0.77 (0.33-1.81)	0.46 (0.14-1.53)

Abbreviations: ACM, all-cause mortality; ADT, androgen deprivation therapy; HR, hazard ratio; PCSM, prostate cancer–specific mortality; sHR, subdistribution HR.

^a^ Includes 31 events among the 145 patients.

^b^ Includes 22 events among the 145 patients.

**Table 3.** Covariate-Adjusted HRs of Mortality in the 139 Patients With Performance Status of 0

Covariate	End point	
	ACM, HR (95% CI)^a^	PCSM, sHR (95% CI)^b^
With vs without docetaxel	0.46 (0.21-1.02)	0.30 (0.11-0.86)
Clinical T category T3-T4 vs T1-T2 or TX	1.64 (0.78-3.48)	2.32 (1.04-5.22)
Gleason score 9-10 vs 8	2.20 (0.90-5.34)	3.14 (0.78-12.66)
ADT duration long-term vs short-term	0.77 (0.33-1.81)	0.46 (0.14-1.53)

Abbreviations: ACM, all-cause mortality; ADT, androgen deprivation therapy; HR, hazard ratio; PCSM, prostate cancer–specific mortality; sHR, subdistribution HR.

^a^ Includes 27 events among the 139 patients.

^b^ Includes 19 events among the 139 patients.

### Unadjusted Estimates of ACM and PCSM

**Figure 2.**
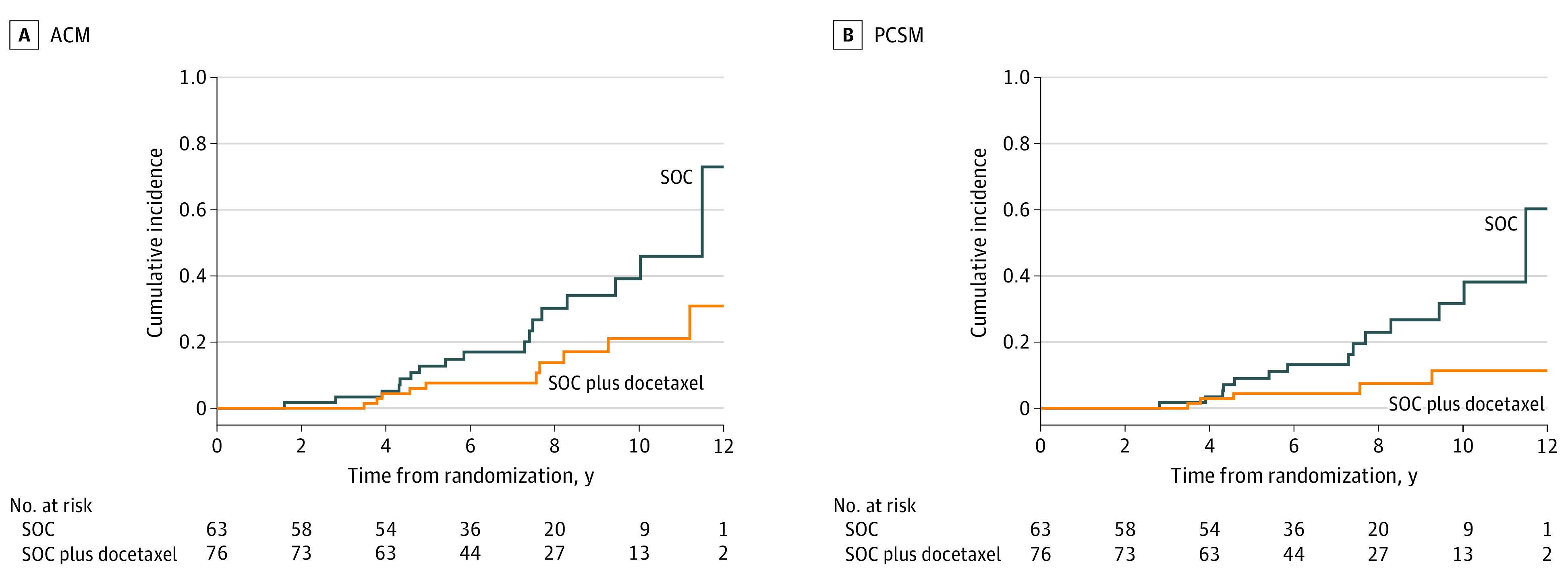
Unadjusted Cumulative Incidence of All-Cause Mortality (ACM) and Cumulative Incidence Function of Prostate Cancer–Specific Mortality (PCSM) After Accounting for Competing Risk Includes the 139 patients with performance status of 0, comparing patients in the standard of care (SOC) group with those in the SOC plus docetaxel group. For ACM, log-rank *P* = .03. For PCSM, Gray *P* = .007.

To illustrate the results of the models for patients with performance status of 0, Figure 2 depicts the unadjusted estimates of ACM and PCSM for patients randomized to SOC plus docetaxel vs SOC. After adjustment for multiple comparisons (ie, all 145 patients and the 139 patients with performance status of 0; 2-sided *P* < .025 was considered statistically significant), the comparisons of the estimates between randomized treatment groups was not significant for ACM (*P* = .03) and was significant for PCSM (*P* = .007). Specifically, among the 139 patients with performance status of 0, the unadjusted 8-year ACM estimate (Figure 2A) was 30% (95% CI, 18%-48%) for SOC vs 14% (95% CI, 6%-28%) for SOC plus docetaxel; the unadjusted 8-year PCSM estimate (Figure 2B) was 23% (95% CI, 11%-38%) for SOC vs 8% (95% CI, 2%-18%) for SOC plus docetaxel.

## Discussion

In this meta-analysis, we found that patients with localized or locally advanced nonmetastatic, high-grade prostate cancer with low PSA levels (<4 ng/mL) had a nonsignificant reduction in the risk of both PCSM and ACM when docetaxel was added to the SOC therapies of testosterone suppression plus radiotherapy or RP. This observation was also valid but was more pronounced among patients with performance status of 0, for whom the reduction in PCSM risk was significant. The clinical importance of these findings is that in this patient cohort with progression to a castrate-resistant state early in the disease course,^7^ both PCSM and ACM risk may be reduced with the addition of docetaxel to testosterone suppression plus radiotherapy or RP.

Several points require further discussion. First, the performance status was associated with both PCSM and ACM. While the latter can be explained by competing risks in patients with comorbidity, the former can be explained by comorbidity in patients with performance status of 1, which can lead to a decreased ability to tolerate and complete the prescribed course of PC treatment. Moreover, while both PCSM and ACM were reduced among all men in the study cohort, these associations were more pronounced among men in otherwise good health (performance status of 0), highlighting the importance of performance status on outcomes following docetaxel use. Further study is warranted to assess the effect of docetaxel use on PCSM and ACM among patients with performance status of 1, given that only 6 men in the current study had a performance status of 1. In addition, with only 5 eligible patients in the GETUG-12 RCT,^16^ a sensitivity analysis to assess whether these patients benefited from the addition of docetaxel despite concurrent estramustine use was not possible. Second, while the study cohort included only 6.6% of all patients enrolled in the 4 eligible RCTs, death due to prostate cancer in this patient subgroup was the leading cause of death, accounting for 71.0% of all observed deaths; this finding illustrates the importance of identifying more effective therapy for reducing PCSM that can in turn reduce the risk of ACM. Specifically, by adding docetaxel to the SOC treatment, the absolute PCSM rate decreased more than 2-fold (from 15 to 7) among all patients and nearly 3-fold (from 14 to 5) in patients with a performance status of 0. Third, while the 95% CI included 1.00, numerical reduction occurred in PCSM and ACM among patients who received long-term (ie, 2 years) compared with short-term (ie, ≤6 months) ADT with testosterone suppression therapy. This observation is consistent with findings of a prior RCT^27^ where long-term and short-term ADT were compared among patients with locally advanced prostate cancer. Given that in the RP RCT,^17^ men in the docetaxel group also received a short course (18-24 weeks) of ADT, they would have been included in the 4- to 6-month ADT cohort; given the docetaxel use, this could have reduced PSCM and ACM, thereby decreasing power to assess a reduction in PCSM and ACM risk in patients receiving long-term compared with short-term ADT. Therefore, we believe it is prudent to consider long-term ADT as part of the testosterone suppression treatment plan when using radiotherapy as the local treatment in patients with Gleason scores of 8 to 10 and PSA levels of less than 4 ng/mL.

Finally, a meta-analysis of 2 phase 3 RCTs^30^ has shown that among patients treated with primary radiotherapy, adjuvant hormonal therapy using testosterone plus adrenal androgen suppression with abiraterone acetate has been associated with both a metastasis-free survival (MFS) and OS benefit in very high-risk prostate cancer when compared with testosterone suppression alone. Some patients enrolled in that study are included in the current study; namely those with Gleason scores of 8 to 10 and T3 to T4 prostate cancer. Therefore, it would be of interest to know whether the patient subgroup defined by a PSA level of less than 4 ng/mL, Gleason score of 8 to 10, and T category of T3 to T4 benefited from the addition of abiraterone to testosterone suppression therapy and radiotherapy. If true, then 2 options would exist to improve outcomes in this patient population. Further study would then be indicated in this patient cohort to ascertain whether triple therapy with testosterone suppression, abiraterone, and docetaxel is superior with respect to OS to the doublet of testosterone suppression and abiraterone. In addition, several androgen receptor–signaling inhibitors have been shown to prolong MFS in patients with castrate-resistant or ADT-resistant nonmetastatic prostate cancer.^31,32,33^ Multiple investigations studying the treatment effect on OS of adding these agents to testosterone suppression treatment with radiotherapy or RP in men with high-risk prostate cancer are actively recruiting or are in follow-up.^34,35^ The treatment effect of these more potent hormonal therapies in patients with high-risk prostate cancer and a PSA level of less than 4 ng/mL and Gleason score of 8 to 10 will be of major interest. If an OS benefit is observed with addition of the more potent hormonal therapy in this patient subgroup, then multiple options for treatment escalation will exist. Further study would be needed to determine the optimal specific combinations and sequencing of these agents for patients with a PSA level of less than 4 ng/mL and Gleason score of 8 to 10 and what effect docetaxel has on OS when added to these agents. It is also possible that a luminal proliferating gene expression profile or other genomic classifiers may be able identify which patients are most likely to benefit from docetaxel.^36,37^

### Limitations

Limitations of this study exist. First, 94 of the 145 patients (64.8%) had local treatment with RP, and these patients could represent a distinct subset, for example with less underlying comorbidity, compared with those who underwent radiotherapy. Second, 38 patients (26.2%) were in trials where 2 years of ADT was used. While we adjusted for ADT duration in the model and docetaxel use was associated with a 70% reduction in PCSM risk among patients with performance status of 0, the question remains as to what that result would have been if all patients received 2 years of ADT. Third, with 31 deaths representing only 21.4% of the study cohort, the data are still maturing. In addition, it is unlikely that an RCT addressing this question will be performed in this patient group with a poor prognosis. As a result, the current data are all that are available to address this question. Therefore, as the data mature, more precise estimates of the possible benefit of adding docetaxel to SOC in patients with a performance status of 0 will be needed before routine adoption into clinical practice. Metastasis-free survival has been shown to be a surrogate for overall survival.^38^ However, MFS was not a primary end point in the RCTs evaluated in the current study.^13,14,17,18^ As a result, the MFS results may be subject to ascertainment bias and not be reliable, given that guidelines on when to look for metastasis and by what means were not prespecified. Fourth, given only 6 patients with a performance status of 1, an interaction test between performance status and the docetaxel treatment effect was not possible. Therefore, the reductions in PCSM and ACM risk may be similar among patients with performance status of 0 and 1. Finally, the inability to report a measure of heterogeneity because 1 RCT^14^ only provided 9 patients to the study cohort raises the possibility of overstating the precision of results. This possibility exists because 1 of the 4 RCTs included in the meta-analysis was the source trial^18^ that generated the hypothesis of potential benefit when adding docetaxel to SOC treatment.

## Conclusions

In this meta-analysis of RCTs, we found that adding docetaxel to SOC treatment in patients with prostate cancer who are in otherwise good health with a PSA level of less than 4 ng/mL and a Gleason score of 8 to 10 was associated with a significant reduction in PCSM and therefore has the potential to improve prognosis. In the future, specific attention should be given to this patient subgroup with poor prognosis when reporting on studies that evaluate the effect of more potent hormonal therapies on OS.

## Notes

Supplement.
Data Sharing Statement

## References

[1] D’Amico AV, Chen M, Malkowicz SB, . Lower prostate specific antigen outcome than expected following radical prostatectomy for patients with high grade prostate cancer and a PSA level of 4 ng/ml or less. J Urol. 2002;167:2025-2031. doi:10.1016/S0022-5347(05)65076-8 11956431

[2] Kim DW, Chen MH, Wu J, . Prostate-specific antigen levels of ≤4 and >4 ng/mL and risk of prostate cancer-specific mortality in men with biopsy Gleason score 9 to 10 prostate cancer. Cancer. 2021;127(13):2222-2228. doi:10.1002/cncr.33503 34101827

[3] Falchook AD, Martin NE, Basak R, Smith AB, Milowsky MI, Chen RC. Stage at presentation and survival outcomes of patients with Gleason 8-10 prostate cancer and low prostate-specific antigen. Urol Oncol. 2016;34(3):119.e19-119.e26. doi:10.1016/j.urolonc.2015.09.014 26526383

[4] Sandblom G, Ladjevardi S, Garmo H, Varenhorst E. The impact of prostate-specific antigen level at diagnosis on the relative survival of 28 531 men with localized carcinoma of the prostate. Cancer. 2008;112(4):813-819. doi:10.1002/cncr.23235 18098207

[5] Fankhauser CD, Penney KL, Gonzalez-Feliciano AG, . Inferior cancer survival for men with localized high-grade prostate cancer but low prostate-specific antigen. Eur Urol. 2020;78(4):637-639. doi:10.1016/j.eururo.2020.05.035 32624279 PMC8411278

[6] Fankhauser CD, Parry MG, Ali A, . A low prostate specific antigen predicts a worse outcome in high but not in low/intermediate-grade prostate cancer. Eur J Cancer. 2023;181:70-78. doi:10.1016/j.ejca.2022.12.017 36641896

[7] Mahal BA, Yang DD, Wang NQ, . Clinical and genomic characterization of low-prostate-specific antigen, high-grade prostate cancer. Eur Urol. 2018;74(2):146-154. doi:10.1016/j.eururo.2018.01.043 29478736 PMC6615042

[8] Petrylak DP, Tangen CM, Hussain MH, . Docetaxel and estramustine compared with mitoxantrone and prednisone for advanced refractory prostate cancer. N Engl J Med. 2004;351(15):1513-1520. doi:10.1056/NEJMoa041318 15470214

[9] Tannock IF, de Wit R, Berry WR, ; TAX 327 Investigators. Docetaxel plus prednisone or mitoxantrone plus prednisone for advanced prostate cancer. N Engl J Med. 2004;351(15):1502-1512. doi:10.1056/NEJMoa040720 15470213

[10] Sweeney CJ, Chen YH, Carducci M, . Chemohormonal therapy in metastatic hormone-sensitive prostate cancer. N Engl J Med. 2015;373(8):737-746. doi:10.1056/NEJMoa1503747 26244877 PMC4562797

[11] Clarke NW, Ali A, Ingleby FC, . Addition of docetaxel to hormonal therapy in low- and high-burden metastatic hormone sensitive prostate cancer: long-term survival results from the STAMPEDE trial. Ann Oncol. 2019;30(12):1992-2003. doi:10.1093/annonc/mdz396 31560068 PMC6938598

[12] Lin DW, Shih MC, Aronson W, . Veterans Affairs Cooperative Studies Program Study #553: chemotherapy after prostatectomy for high-risk prostate carcinoma: a phase III randomized study. Eur Urol. 2020;77(5):563-572. doi:10.1016/j.eururo.2019.12.020 31924316

[13] Sartor O, Karrison TG, Sandler HM, . Androgen deprivation and radiotherapy with or without docetaxel for localized high-risk prostate cancer: long-term follow-up from the randomized NRG Oncology RTOG 0521 trial. Eur Urol. 2023;84(2):156-163. doi:10.1016/j.eururo.2023.04.024 37179241 PMC10662642

[14] James ND, Sydes MR, Clarke NW, ; STAMPEDE investigators. Addition of docetaxel, zoledronic acid, or both to first-line long-term hormone therapy in prostate cancer (STAMPEDE): survival results from an adaptive, multiarm, multistage, platform randomised controlled trial. Lancet. 2016;387(10024):1163-1177. doi:10.1016/S0140-6736(15)01037-5 26719232 PMC4800035

[15] Kellokumpu-Lehtinen PL, Hjälm-Eriksson M, Thellenberg-Karlsson C, ; Investigators of the Scandinavian Prostate Cancer Study No. 13. Docetaxel versus surveillance after radical radiotherapy for intermediate- or high-risk prostate cancer—results from the prospective, randomised, open-label phase III SPCG-13 trial. Eur Urol. 2019;76(6):823-830. doi:10.1016/j.eururo.2019.08.010 31443961

[16] Fizazi K, Faivre L, Lesaunier F, . Androgen deprivation therapy plus docetaxel and estramustine versus androgen deprivation therapy alone for high-risk localised prostate cancer (GETUG 12): a phase 3 randomised controlled trial. Lancet Oncol. 2015;16(7):787-794. doi:10.1016/S1470-2045(15)00011-X 26028518

[17] Eastham JA, Heller G, Halabi S, . Cancer and Leukemia Group B 90203 (Alliance): radical prostatectomy with or without neoadjuvant chemohormonal therapy in localized, high-risk prostate cancer. J Clin Oncol. 2020;38(26):3042-3050. doi:10.1200/JCO.20.00315 32706639 PMC7479762

[18] D’Amico AV, Xie W, McMahon E, . Radiation and androgen deprivation therapy with or without docetaxel in the management of nonmetastatic unfavorable-risk prostate cancer: a prospective randomized trial. J Clin Oncol. 2021;39(26):2938-2947. doi:10.1200/JCO.21.00596 34197181 PMC8425842

[19] Schaeffer E, Srinivas S, Antonarakis ES, . NCCN Guidelines insights: prostate cancer, version 1.2021. J Natl Compr Canc Netw. 2021;19(2):134-143. doi:10.6004/jnccn.2021.0008 33545689

[20] D’Amico AV, Chen MH, Renshaw AA, Loffredo M, Kantoff PW. Androgen suppression and radiation vs radiation alone for prostate cancer: a randomized trial. JAMA. 2008;299(3):289-295. doi:10.1001/jama.299.3.289 18212313

[21] Lubiniecki GM, Berlin JA, Weinstein RB, Vaughn DJ. Thromboembolic events with estramustine phosphate–based chemotherapy in patients with hormone-refractory prostate carcinoma: results of a meta-analysis. Cancer. 2004;101(12):2755-2759. doi:10.1002/cncr.20673 15536625

[22] El K, Meier P. Nonparametric estimation from incomplete observations. J Am Stat Assoc. 1958;53:457-481. doi:10.1080/01621459.1958.10501452

[23] Gray RJ. A class of K-sample tests for comparing the cumulative incidence of a competing risk. Ann Stat. 1988;16:1141-1154. doi:10.1214/aos/1176350951

[24] Klein J, Moeschberger M. Survival Analysis: Techniques for Censored and Truncated Data. Springer; 2013.

[25] Cox D. Regression models and life tables. J R Stat Soc B. 1972;34(2):187-220.

[26] Fine JP, Gray RJ. A proportional hazards model for the subdistribution of a competing risk. J Am Stat Assoc. 1999;94:496-509. doi:10.1080/01621459.1999.10474144

[27] Bolla M, de Reijke TM, Van Tienhoven G, ; EORTC Radiation Oncology Group and Genito-Urinary Tract Cancer Group. Duration of androgen suppression in the treatment of prostate cancer. N Engl J Med. 2009;360(24):2516-2527. doi:10.1056/NEJMoa0810095 19516032

[28] Kutner M, Nachtshein CJ, Neter J, Li W. Applied Linear Statistical Models. 5th ed. McGraw Hill/Irwin; 2005.

[29] Schemper M, Smith TL. A note on quantifying follow-up in studies of failure time. Control Clin Trials. 1996;17(4):343-346. doi:10.1016/0197-2456(96)00075-X 8889347

[30] Attard G, Murphy L, Clarke NW, ; Systemic Therapy in Advancing or Metastatic Prostate cancer: Evaluation of Drug Efficacy (STAMPEDE) investigators. Abiraterone acetate and prednisolone with or without enzalutamide for high-risk non-metastatic prostate cancer: a meta-analysis of primary results from two randomised controlled phase 3 trials of the STAMPEDE platform protocol. Lancet. 2022;399(10323):447-460. doi:10.1016/S0140-6736(21)02437-5 34953525 PMC8811484

[31] Smith MR, Saad F, Chowdhury S, ; SPARTAN Investigators. Apalutamide treatment and metastasis-free survival in prostate cancer. N Engl J Med. 2018;378(15):1408-1418. doi:10.1056/NEJMoa1715546 29420164

[32] Hussain M, Fizazi K, Saad F, . Enzalutamide in men with nonmetastatic, castration-resistant prostate cancer. N Engl J Med. 2018;378(26):2465-2474. doi:10.1056/NEJMoa1800536 29949494 PMC8288034

[33] Fizazi K, Shore N, Tammela TL, ; ARAMIS Investigators. Darolutamide in nonmetastatic, castration-resistant prostate cancer. N Engl J Med. 2019;380(13):1235-1246. doi:10.1056/NEJMoa1815671 30763142

[34] Chen RC, Karrison T, Lawton CA, . INNOVATE (NRG-GU008): a randomized phase III trial of salvage radiotherapy and androgen deprivation therapy (ADT) with/without abiraterone and apalutamide for patients with node-positive prostate cancer after radical prostatectomy. J Clin Oncol. Published online March 2, 2021. doi:10.1200/JCO.2021.39.6_suppl.TPS179

[35] Nguyen PL, Sartor AO. Parallel phase III randomized trials for high risk prostate cancer evaluating de-intensification for lower genomic risk and intensification of concurrent therapy for higher genomic risk with radiation (Predict-RT*) *prostate RNA expression/decipher to individualize concurrent therapy with radiation. Accessed October 3, 2023. https://www.nrgoncology.org/Clinical-Trials/Protocol/nrg-gu009-1?filter=nrg-gu009-1

[36] Hamid AA, Huang HC, Wang V, . Transcriptional profiling of primary prostate tumor in metastatic hormone-sensitive prostate cancer and association with clinical outcomes: correlative analysis of the E3805 CHAARTED trial. Ann Oncol. 2021;32(9):1157-1166. doi:10.1016/j.annonc.2021.06.003 34129855 PMC8463957

[37] Phillips R, . Basal-luminal subtyping of localized high-risk prostate cancer and benefit of adding docetaxel to definitive radiotherapy with androgen suppression in the NRG Oncology/RTOG 0521 phase III trial. J Clin Oncol. 2023;41(16)(suppl):5094. doi:10.1200/JCO.2023.41.16_suppl.5094 PMC12370384 40675873

[38] Xie W, Regan MM, Buyse M, ; ICECaP Working Group. Metastasis-free survival is a strong surrogate of overall survival in localized prostate cancer. J Clin Oncol. 2017;35(27):3097-3104. doi:10.1200/JCO.2017.73.9987 28796587 PMC5652387

