# Supplementary material for: Mortality Risk for Docetaxel-Treated, High-Grade Prostate Cancer With Low PSA Levels: A Meta-Analysis
Source: JAMA Netw Open. 2023 Nov 1;6(11):e2340787. doi: 10.1001/jamanetworkopen.2023.40787 (PMC10620614; doi:10.1001/jamanetworkopen.2023.40787)
Supplement: Supplement. — Data Sharing Statement [file jamanetwopen-e2340787-s001.pdf]

Data Sharing Statement

Mahal. Mortality Risk for Docetaxel-Treated, High-Grade Prostate Cancer With Low PSA Levels. *JAMA Netw Open*. Published November 01, 2023.
doi:10.1001/jamanetworkopen.2023.40787

Data
Data available: No

Additional Information
Explanation for why data not available: Any investigator can request data from the ICECaP consortium data base by contacting Wanling Xie and Christopher Sweeney and Praful Ravi (co-authors on the current study)
